# Inhibition of p70S6K2 down-regulates Hedgehog/GLI pathway in non-small cell lung cancer cell lines

**DOI:** 10.1186/1476-4598-8-44

**Published:** 2009-07-06

**Authors:** Shinji Mizuarai, Aki Kawagishi, Hidehito Kotani

**Affiliations:** 1 Departments of Oncology, Tsukuba Research Institute, Merck Research Laboratories, Banyu Pharmaceutical Co, Ltd, Tsukuba, Ibaraki 300-2611, Japan; 2 Corporate services, Banyu Pharmaceutical Co, Ltd, Chiyoda-Ku, Tokyo 102-8667, Japan

## Abstract

**Background:**

The Hedgehog (HH) pathway promotes tumorigenesis in a diversity of cancers. Activation of the HH signaling pathway is caused by overexpression of HH ligands or mutations in the components of the HH/GLI1 cascade, which lead to increased transactivation of GLI transcription factors. Although negative kinase regulators that antagonize the activity of GLI transcription factors have been reported, including GSK3β, PKA and CK1s, little is known regarding positive kinase regulators that are suitable for use on cancer therapeutic targets. The present study attempted to identify kinases whose silencing inhibits HH/GLI signalling in non-small cell lung cancer (NSCLC).

**Results:**

To find positive kinase regulators in the HH pathway, kinome-wide siRNA screening was performed in a NSCLC cell line, A549, harboring the GLI regulatory reporter gene. This showed that p70S6K2-silencing remarkably reduced GLI reporter gene activity. The decrease in the activity of the HH pathway caused by p70S6K2-inhibition was accompanied by significant reduction in cell viability. We next investigated the mechanism for p70S6K2-mediated inhibition of GLI1 transcription by hypothesizing that GSK3β, a negative regulator of the HH pathway, is activated upon p70S6K2-silencing. We found that phosphorylated-GSK3β (Ser9) was reduced by p70S6K2-silencing, causing a decreased level of GLI1 protein. Finally, to further confirm the involvement of p70S6K2 in GLI1 signaling, down-regulation in GLI-mediated transcription by PI3KCA-inhibition was confirmed, establishing the pivotal role of the PI3K/p70S6K2 pathway in GLI1 cascade regulation.

**Conclusion:**

We report herein that inhibition of p70S6K2, known as a downstream effector of the PI3K pathway, remarkably decreases GLI-mediated transactivation in NSCLC by reducing phosphorylated-GSK3β followed by GLI1 degradation. These results infer that p70S6K2 is a potential therapeutic target for NSCLC with hyperactivated HH/GLI pathway.

## Background

The Hedgehog (HH) signaling pathway is essential for the control of multiple cell proliferation processes such as pattern formation, stem cell maintenance and tumorigenesis [1,2]. Activation of HH signaling is initiated by the HH ligand binding to its receptor, Patched (PTCH), leading to relief of PTCH mediated repression of a G protein-coupled receptor, Smoothened (SMOH) [3]. This event is followed by the accumulation of unphosphorylated GLI transcription factors at multiple amino acid residues [4]. The hypophosphorylation of GLI causes its stabilization, which facilitates the transactivation of GLI regulatory genes involved in cell cycle progression and apoptosis inhibition such as *Cyclin D1 *[5], *γ-catenin *[6], and self-induction of *GLI1 *[7]. The eventual transactivation/transsuppression of a number of genes by GLI transcription factors is of significance for exertion of the HH signaling cascade's functions in normal-cell development or tumorigenesis. The regulation of HH signaling is controlled by the conserved negative kinase regulators, protein kinase A (PKA), casein kinases (CK1a and CK1e) and glycogen synthase kinase 3β (GSK3β) which cooperatively phosphorylate and inactivate GLI factors [8-10]. Up-regulation of *PTCH *expression by HH signaling is also an important feature of negative feedback [7]. Positive regulation is controlled by the feedback loop of GLI transcription factors which directly induce expression via binding to their promoters [7]. Although the mechanism for coordinated regulation of GLI mediated transcription by HH ligands and downstream positive and negative effectors has been progressively investigated, further analysis to decipher the components involved in the HH cascade is eagerly anticipated.

Along with the multiple cellular processes and functions known to be derived from HH cascade activation, recent findings showing that the HH pathway plays a pivotal role in stem cell maintenance have attracted great attention, especially in the field of cancer research as a new potential therapeutic target pathway for the treatment of various types of cancers [5,11,12]. The aberrant up-regulation of the HH pathway in tumorigenesis was first reported in basal cell carcinomas resulting from either loss-of-function mutation in PTCH [13,14] or gain-of-function mutation in SMOH [15]. The mutations or deregulated expression in PTCH and SMOH have been subsequently reported in various studies of brain, skin and muscle cancers [16,17], which are now categorized as ligand-independent HH cascade-activated cancers. Recently, a subset of non-small cell lung cancer (NSCLC) was found to be hyperactive in the HH/GLI pathway independent of the ligands by expressing high level of GLI1 protein [18]. The other type of cancer in which the HH pathway is up-regulated is ligand-dependent cancer, including prostate cancer [19], breast cancer [20], pancreatic carcinoma [21], and small cell lung carcinoma [22]. The evidence provided in these studies that the HH pathway is activated in a wide range of cancers suggests the importance of identification of effective therapeutic targets to interfere with the HH pathway [23]. For ligand independent cancers there is a particularly urgent need to find effective targets to suppress the GLI cascade due to the ineffectiveness of SMOH inhibitors and other modalities to inhibit upstream components of the HH/GLI cascade [18].

p70S6K2 is a member of the ribosomal S6 kinase family and is involved in protein synthesis and cell proliferation [24,25]. Increased activity or overexpression of p70S6K1/2 has been reported in several types of cancers [26-28]. p70S6K2 is known to mainly work downstream of the phosphoinositide 3-kinase (PI3K) pathway [29,30]. Up-regulation of PI3K signaling by the activating mutation in PI3K; the inactivating mutation in phosphatase and tensin homolog (PTEN); or, receptor tyrosine kinase (RTK)s activation through mitogenic stimuli, results in an increase in serine-threonine kinase AKT activity, which leads to the inactivating phosphorylation of tuberin (TSC), and the activation of mammalian target of rapamycin (mTOR) [29,31]. The increased activity of mTOR drives the subsequent activation of its effectors including p70S6K1/2 and 4E-BP1 [27]. The phosphorylated and activated forms of p70S6K2 and 4E-BP1 cooperatively promote translational up-regulation of the proteins needed for cell cycle promotion. The functional role of p70S6K1/2 in the PI3K/mTOR cascade has been well established in the vast majority of cancer and development research [29-31], and the role of p70S6K inhibition in suppressing PI3K pathway-activated cancers has been extensively studied. However, the involvement of p70S6K in the regulation of the HH signaling pathway has not been analyzed.

In this study, a kinome-wide siRNA screen was performed to identify kinases whose silencing inhibits HH/GLI signaling in NSCLC. We found that *p70S6K2*-silencing by siRNA decreases GLI regulatory transcription ability in NSCLC through modulating GSK3β. This report provides the first evidence that p70S6K2 positively regulates the HH cascade and could serve as a therapeutic target in GLI1 cascade-activated NSCLC independent of HH ligands.

## Results

### Kinome small interfering RNA (siRNA) screening to find Hedgehog (HH) pathway regulatory kinases

**Figure 1:**
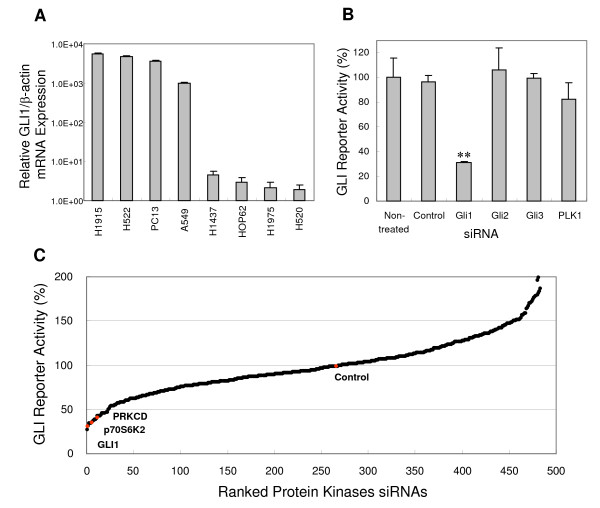
**Kinome-wide siRNA screen to find HH pathway regulatory kinases in NSCLC A549 cells**. A, Identification of GLI1 cascade activated NSCLC cell lines. The mRNA expression level of *GLI1 *in the eight NSCLC cells was measured with quantitative RT-PCR. Data were normalized to internal β-actin expression and are presented as the mean and SD of three independent experiments. B, Establishment of stable A549 cell lines harboring GLI regulatory *β-lactamase *reporter gene activity. The A549-GLI cells were treated with siRNAs as a control, *GLI1, GLI2, GLI3 *or *PLK1 *to confirm that β-lactamase was under the control of GLI1 transcription factor. *PLK1*-siRNA was used as a negative control to confirm that cytotoxicity did not affect reporter activity. Data are the mean and SD of three independent experiments. C, Results of the kinome-wide siRNA screen composed of about 500 protein kinases. The GLI reporter activity was shown as a percentage normalized to control-siRNA treated cells. **, *P *≤ 0.01, compared with control.

It has previously been reported that the HH/GLI1 pathway is activated in some portion of NSCLC cell lines and primary lung tumors [18]. Expression of GLI1 transcription factor, which is a surrogate index of HH/GLI1 activation level, was examined in a panel of NSCLC cells lines to find a suitable cell line for a kinome-wide small interfering RNA (siRNA) screen. Consistent with previous studies, it was found that various levels of *GLI1 *were expressed in the cell lines, indicating that the HH/GLI1 pathway plays a pivotal role in NSCLC cancer cell progression (Fig. 1A). Of the eight cell lines examined, four showed activated HH/GLI1 pathways (A549, H522, PC13 and H1915). Of these, A549 was chosen for the subsequent kinome-siRNA screen, as the status of cancer-related pathways in A549 cells has been well characterized, and A549 cells are amenable to sufficient siRNA transfection. GLI-regulatory *β-lactamase *reporter gene was transferred to the A549 cells and stable cell lines constitutively expressing the reporter gene were established (A549-GLI). Examination of reporter activity after introduction of *GLI1*-siRNA into the A549-GLI cells to confirm that the GLI regulatory *β-lactamase *reporter gene was under the control of GLI1 transcription factor, showed that reporter gene activity was reduced to 32% compared with control-siRNA treated cells (Fig. 1B). The silencing of GLI3, known to be a transcriptional repressor of GLI-regulatory target genes, did not affect β-lactamase activity, indicating that the prominently over-expressed GLI1 in A549 is a major regulator of the *β-lactamase *reporter gene. This suggests that A549-GLI cells were well suited to the kinome-wide siRNA screen to identify kinases that influence HH/GLI1 pathway-mediated transcription. To find kinases that affect the GLI regulatory reporter gene, the A549-GLI cells were transfected by lipofection method with kinome-siRNAs comprising about 500 protein kinases. β-lactamase activity was measured 72 hr after transfection to examine the inhibitory effect on the reporter gene by each kinase (Fig. 1C). In the large scale siRNA screen, *GLI1*-siRNA was also included as a positive control, and about 70% inhibition of reporter activity was constantly observed by the GLI1 disruption, demonstrating that accuracy/reproducibility of the assay were reliable. The result of the siRNA screen illustrated that 17 kinases out of 500 siRNAs reduced the GLI-mediated reporter gene activity to less than 45%. As protein kinase C delta (PRKCD) was previously reported to positively regulate HH/GLI1 pathway [32], kinase siRNAs that down-regulated the reporter activity more than the cutoff value (45%), which was determined based on the reduction level for *PRKCD*-siRNA, were selected as promising candidates as positive regulators for HH/GLI1 pathway. Among the kinase siRNAs that were hit, *p70S6K2 *(*RPS6KB2*) significantly reduced GLI-mediated reporter gene transcription activity to 38%. Although it is well recognized that inhibition of p70S6K2 down-regulates the oncogenic PI3K pathway, the effect of p70K6K2 on the activity of the HH pathway has not been reported. Therefore, we focused on p70S6K2 in the subsequent confirmation and validation studies.

### Inhibition of p70S6K2 reduces GLI1 regulatory transcription

**Figure 2:**
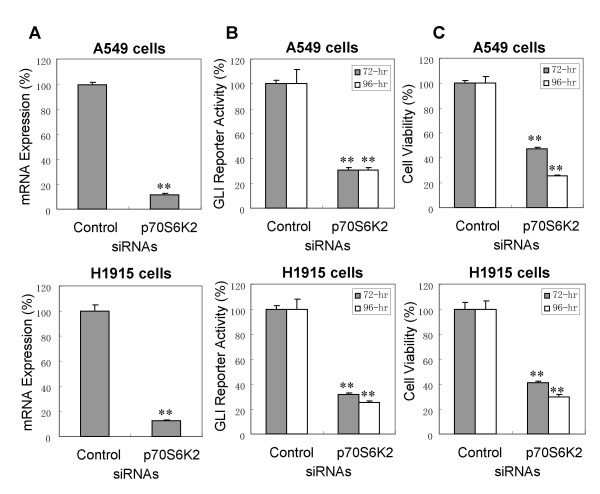
**p70S6K2-silencing by siRNA down-regulates GLI regulatory reporter gene**. A, mRNA-silencing level of *p70S6K2 *by siRNA. mRNA expression levels of *p70S6K2 *in the control- or *p70S6K2*-siRNA treated cells were measured 48 hr after siRNA transfection by quantitative RT-PCR. B, Effect of *p70S6K2*-silencing on GLI reporter gene activity. GLI regulatory β-lactamase activity was measured 72 and 96 hr after siRNA transfection. The value was normalized to cell number. C, Effect of *p70S6K2*-silencing on cell viability. Cell viability was measured 72 and 96 hr after siRNA transfection as described in the materials and methods. Data are the mean and SD of three independent experiments. **, *P *≤ 0.01, compared with control.

**Figure 3:**
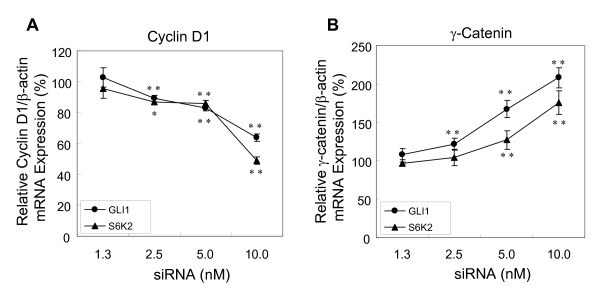
**p70S6K2-silencing by siRNA decreases or increases expression of endogenous GLI1 regulatory genes**. A, *Cyclin D1 *mRNA expression level. B, *γ-catenin *mRNA expression level. mRNA expression levels of GLI1-regulatory genes were measured 48 hr after siRNA transfection by quantitative RT-PCR in A549 cells treated with *GLI1*-siRNA or p*70S6K2*-siRNA. Data were normalized to internal β-actin expression and are presented as the mean and SD of three independent experiments. *, *P *≤ 0.05; **, *P *≤ 0.01 compared with control.

The confirmation studies verified the down-regulation of GLI1 transcription by *p70S6K2 *inhibition. Treatment of A549-GLI with a different sequence of *p70S6K2*-siRNA from the one used in the large scale siRNA screen, followed by recovery of RNA from the transfected cells 48-hr after siRNA transfection, and measurement of the silencing level of *p70S6K2 *by quantitative reverse transcriptase-polymerase chain reaction (RT-PCR) (Fig. 2A) showed that *p70S6K2 *mRNA expression was reduced to 11% compared with control-siRNA treated cells. This illustrates that sufficient level of repression was achieved for *p70S6K2*. Similar to the large scale siRNA screen, 69% reduction in GLI regulatory reporter gene activity was observed at 72 and 96 hr after transfection (Fig. 2B), which was equivalent to the reduction level achieved by *GLI1*-siRNA. Measurement of cell viability after *p70S6K2 *inhibition by quantifying ATP level as an index of metabolically vital cells allowed examination of whether proliferation of A549 cells was dependent on the GLI1 pathway. A reduction in cell viability of about 50% and 70% was observed 72 and 96 hr respectively after transfection (Fig. 2C). Reduction in both GLI reporter gene activity and cell viability by *p70S6K2*-silencing was also confirmed in H1915 cells that stably expressed GLI regulatory *β-lactamase *gene (Fig. 2A–2C bottom panels). In addition to the GLI regulatory *β-lactamase *reporter gene, expressions of endogenous GLI1 regulatory genes were quantified by RT-PCR. *Cyclin D1*, part of G1/S cell cycle machinery, is known to be mainly controlled by GLI1 and GLI2 in HH pathway activated cells [5]. The expression of *γ-catenin*, which is involved in apoptosis, is repressed by GLI1 transcription factor [6]. Both *cyclin D1 *and *γ-catenin *were significantly down- or up-regulated respectively by the increasing concentration of *p70S6K2*-siRNA (Fig. 3A and 3B). The expression changes caused by the inhibition of *p70S6K2 *were similar to those caused by *GLI1 *inhibition: 50% reduction of *Cyclin D1 *and 1.7-fold induction of *γ-catenin*.

### p70S6K2-silencing degrades GLI1 transcription via activating GSK3β

**Figure 4:**
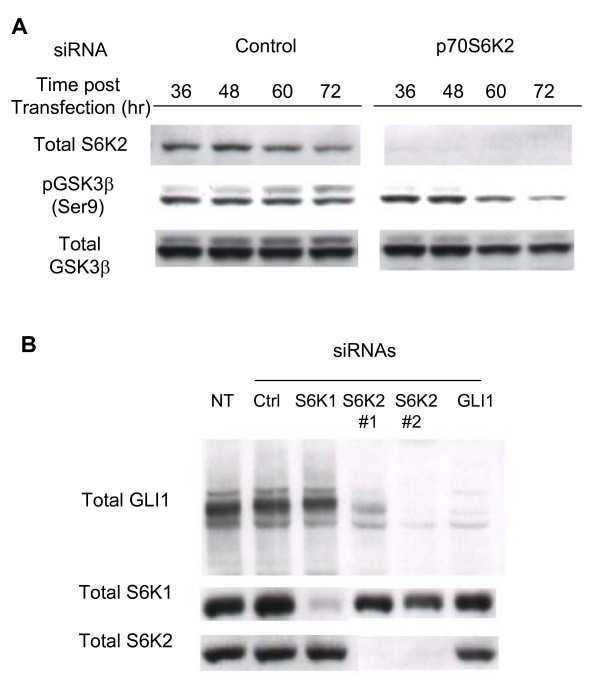
**Inhibition of p70S6K2 causes GLI1 degradation through reduction in phospho-GSK3β at Ser9 residue**. A, Phosphorylated-GSK3β level of cells treated with *p70S6K2 *siRNA. Cell lysates were recovered and subjected to western blotting analysis to detect total p70S6K2, total GSK3β and phospho-GSK3β respectively at the indicated time points after siRNA transfection. B, GLI1 protein level of cells treated with *p70S6K2*-siRNA. A549 cells were also treated with siRNAs (control, *p70S6K1*, *p70S6K2*, and *GLI1*), and cell lysate was subjected to western blotting with GLI1, p70S6K1 and p70S6K2 antibodies 72 hr after transfection.

GSK3β phosphorylates GLI and negatively modulates its activity, leading to the destabilization of the transcription factor. p70S6Ks down-regulates the activity of GSK3β by phosphorylating Ser9 residue [33]. It was hypothesized that the mechanism underlying the p70S6K2 inhibition-mediated down-regulation of GLI1 transcription activity is through the activation of GSK3β which leads to GLI destabilization/inactivation. To examine this hypothesis, phosphorylation levels of GSK3β at Ser9 residue after *p70S6K2*-silencing by siRNA in A549 cells were measured. Through western blotting, it was observed that p70S6K2 levels were remarkably reduced (Fig. 4A), which was in accordance with mRNA expression levels shown previously. Although the phosphorylated form of GSK3β (Ser9) was not affected by control-siRNA treatment, the level of phospho-GSK3β was significantly reduced upon the treatment of *p70S6K2*-siRNA in a time-dependent manner (Fig. 4A). Total GSK3β was also unaltered by the siRNA transfection. As GLI1 is stabilized by the inactivated form of phosporylated-GSK3β, GLI1 protein level was investigated by western blotting when *p70S6K2 *was silenced. Upon the silencing of *p70S6K2 *by siRNA, significant reduction of GLI1 protein level was observed compared with the control (Fig. 4B).

**Figure 5:**
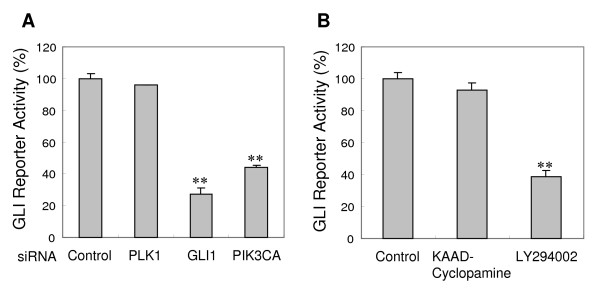
**GLI reporter gene activity reduction by PIK3CA-inhibition**. A, Effect of *PI3K*-silencing by siRNA on GLI regulatory reporter gene. GLI regulatory reporter gene activity was measured by β-lactamase assay 72 hr after siRNA transfection (*PLK1*, *GLI1 *and *PIK3CA*) into A549-GLI cells. *PLK1*-siRNA was treated as a negative control to confirm that cytotoxicity did not affect reporter activity. *GLI1*-siRNA was used as a positive control. B, Effect of PI3K-inhibition by LY294002 on GLI regulatory reporter gene. The A549-GLI cells were treated with PI3K inhibitor, LY294002 (90 μM) and SMOH inhibitor KAAD-cyclopamine (800 nM) respectively. β-lactamase activity was measured 12 hr after treatment. Data are the mean and SD of three independent experiments. **, *P *≤ 0.01 compared with control.

P70S6K2 is well recognized as a downstream effector of the PI3K pathway [27,29], and no relationship between p70S6K2 and the HH pathway has yet been reported. Therefore, to further support the novel finding that p70S6K2, as one of the components of the PI3K pathway, modulates GLI1 transactivation ability, we examined whether phosphatidylinositol 3-kinase catalytic alpha polypeptide (PIK3CA) inhibition reduces GLI regulatory reporter gene activity. In agreement with the *p70S6K2 *inhibition-mediated reduction in the reporter gene, *PIK3CA *silencing by siRNA also decreased GLI regulatory reporter gene activity to 44% in A549-GLI cells (Fig. 5A). The effect of pharmacological inhibition of PI3K on the GLI reporter gene was also examined. While SMOH inhibition by cyclopamine did not affect GLI reporter gene activity in accordance with a previous study that showed GLI1 activation is ligand independent in A549 cells [18], a significant decrease in activity was observed by inhibition of PI3K with LY294002 (Fig. 5B).

The results demonstrated so far, which indicate that p70S6K2-inhibition down-regulated GLI1-mediated transcription via regulation of GSK3β function, were predominately investigated in A549 cells. The activation of GSK3β and GLI1 degradation by *p70S6K2*-silencing was also confirmed in the H1915 cell line.

## Discussion

A number of researchers have reported the development of HH/GLI1 cascade inhibitors as a new class of anti-tumor agent. For HH ligand-dependent cancers, pharmacological inhibition of the upstream components of the pathway offers an effective anti-tumor action. Indeed, ligand neutralizing antibodies or cyclopamine (an SMOH inhibitor) in preclinical studies have shown significant progress in regressing tumor development [5,12]. It has been reported, however, that GLI1 signaling is activated in a subset of NSCLC through the mechanism of overexpression of GLI1 transcription factor with no deregulation of PTCH or SMOH [18]. This signaling activation is ligand-independent, given the fact that cyclopamine had little effect on both cell growth and GLI target gene expression in NSCLC cells. In order to suppress the HH pathway, novel therapeutic targets to intervene in the GLI1 cascade in NSCLC need to be identified. As kinases are widely recognized as druggable proteins which are amenable to the development of small molecule chemical inhibitors, a kinome-wide siRNAs screen was performed to identify kinase regulators of the HH pathway. Unexpectedly, silencing of *p70S6K2*, a key regulator of the PI3K pathway, remarkably reduced the activity of GLI regulatory gene, indicating that p70S6K2 may serve as a therapeutic target to inactivate the HH cascade in cancer. The results of this study demonstrate that *p70S6K2*- and *GLI1*-silencing achieved similar levels of suppression of the GLI regulatory reporter gene. This suggests that pharmacological inhibition of p70S6K2 would sufficiently down-regulate the HH/GLI1 cascade in a subpopulation of NSCLCs with GLI1 overexpression.

**Figure 6:**
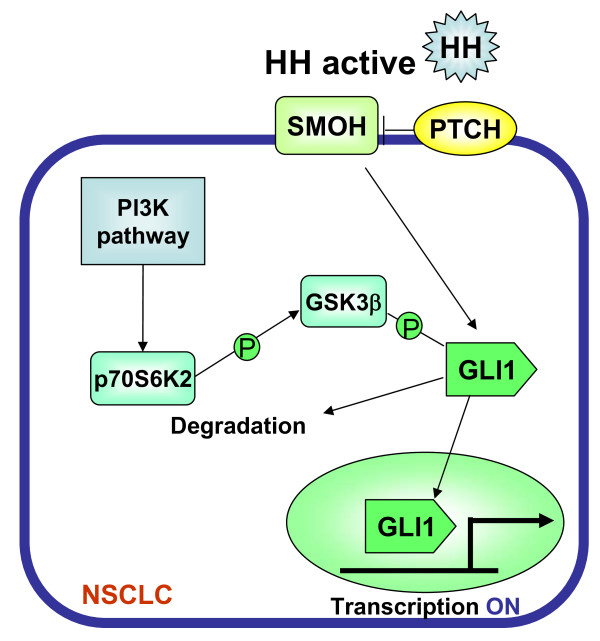
**Proposed model of the relationship between p70S6K2 and the HH pathway**. The activation of the canonical HH pathway is initiated by binding of the HH ligand to its receptor PTCH, which relieves the repression of SMOH. The activation of SMOH is translated into GLI zinc-finger transcription factors. Glycogen synthase kinase-3 beta (GSK3β) negatively regulates GLI transcription factors cooperating with other kinases such as PKA and CK1s. GSK3β is negatively regulated through phosphorylation by p70S6K2 which is a downstream effecter of the PI3K pathway.

The cross-talk between the HH pathway and other cancer relevant pathways has been extensively studied. Stimulation of PRKCD activates ERK signaling and up-regulates GLI transcription without the addition of an HH-ligand [32], indicating the contribution of the PRKCD/ERK pathway to GLI activation. Both activation and inhibition of PRKCD by phorbol esters and pharmacological intervention respectively has illustrated that PRKCD controls GLI activation of HH signaling. The present data from the kinome-wide siRNA screen also identified *PRKCD*-siRNA as a negative regulator of the HH pathway (Table 1); supporting previous evidence that PRKCD functions to control the GLI1cascade. Recent advances in stem cell biology have also presented cross-talk between the HH pathway and other developmental pathways such as Wnt, Hox and Notch signaling [34,35]. Examples include a study on chronic myeloid leukemia stem cells, which showed that HH-dependent Stat3 activation orchestrates down-regulation of Hox genes such as HoxA2 and HoxB4. With respect to the association of HH and PI3K pathways, a pioneering study showed that PI3K itself and AKT were found to be essential for the activation of the GLI reporter gene in response to HH stimulus in non-tumorigenic NIH3T3 cells harboring the GLI regulatory reporter gene, the expression of which is regulated in an HH ligand dependent manner [36]. The same study also indicated that up-regulation of the GLI reporter gene by PI3K/AKT activation is mediated by controlling PKA activity. Singh and colleagues also reported that PI3K/AKT contributes to activation of the HH/GLI1 signaling pathway in ALK-positive anaplastic large cell lymphoma (ALCL), but not in ALK-negative ALCL [37]. However, the involvement of p70S6 kinases was not investigated. The current study provides additional evidence that the PI3K pathway contributes to the activation of the GLI1 cascade in NSCLC cells. Moreover, the mechanism relating PI3K to GLI1 regulation observed in this study is novel and distinct from the previous study in that the downstream effector of PI3K pathway, p70S6K2, controls GLI-mediated transcription via phosphorylating GSK3β which regulates GLI1 stabilization (Fig. 6).

**Table 1:** Kinase siRNAs suppressing GLI-mediated transcriptional activity

**Kinases**	**Official Full Name**	**GLI Reporter Activity (%)**
*ADCK5*	aarF domain containing kinase 5	27 ± 0.7
*TNIK*	TRAF2 and NCK interacting kinase	31 ± 2.9
*PIM3*	pim-3 oncogene	34 ± 2.8
*PAK6*	p21 protein (Cdc42/Rac)-activated kinase 6	35 ± 8.6
*DCLK2*	doublecortin-like kinase 2	35 ± 1.7
*ANKK1*	ankyrin repeat and kinase domain containing 1	35 ± 1.4
*PCTK3*	PCTAIRE protein kinase 3	36 ± 4.8
*PRKD2*	protein kinase D2	37 ± 5.3
*p70S6K2*	ribosomal protein S6 kinase, polypeptide 2	38 ± 3.6
*NLK*	nemo-like kinasae	39 ± 1.4
*DKFZP434C131*	unc-51-like kinase 3	40 ± 3.6
*MASTL*	microtubule associated serine/threonine kinase-like	42 ± 2.0
*LATS2*	large tumor suppressor, homolog 2	42 ± 0.5
*KSR2*	kinase suppressor of ras 2	43 ± 0.5
*STK38L*	serine/threonine kinase 38 like	43 ± 2.8
*MLK4*	mixed lineage kinase 4	44 ± 1.6
*PRKCD*	protein kinase C, delta	45 ± 1.1

The kinome-wide siRNA screen of the HH signaling pathway performed in the present study found that *p70S6K2*-silencing suppresses GLI1-regulatory genes, but *p70S6K1*-silencing does not. Subsequent studies also revealed that the mechanism for down-regulation in the GLI1 cascade is caused by *p70S6K2*-silencing. Recent studies have facilitated our understanding that p70S6K1 and p70S6K2 possess redundant and distinct functions in cell signaling transduction [38]. An example of a commonly conserved function is that both p70S6K1/2 kinases transduce the signal in the down stream of the PI3K/mTOR cascade to accelerate protein biosynthesis. In the present siRNA screen, however, *p70S6K1 *was not identified as a GLI1 cascade inhibitory siRNA. The result that *p70S6K1*-siRNA treatment did not affect the HH/GLI1 cascade in NSCLC cells was also carefully confirmed by independent experiments which measured reduction in *p70S6K1 *expression and GLI regulatory reporter gene activity (data not shown). Moreover, simultaneous double-knockdown of *p70S6K1 *and *p70S6K2 *was investigated to examine any synergistic effect on the inhibition of the GLI1 cascade. This resulted in no enhancing effect on the GLI reporter gene by *p70S6K1*-siRNA. This indicates that the role of p70S6K2 in inhibiting the HH pathway may be distinct from that of p70S6K1, although we cannot eliminate the possibility that p70S6K1 may exert similar function in different types of cells. Further studies to examine expression/activation levels of p70S6K1/2 and the GLI1 cascade in diverse types of clinical samples and cell lines would provide some insights on this issue.

## Conclusion

We report herein that p70S6K2 positively regulates GLI1-mediated transcription through modulating GSK3β in NCSLC. Given the recent finding that various types of tumors have deregulations in the HH/GLI cascade independent of the HH ligand, in which modulation of upstream components are less effective [18], it is imperative that novel therapeutic targets for the GLI1 cascade be identified. The identification of p70S6K2 as a positive regulator of GLI-mediated transcription provides an alternative strategy for developing therapeutic agents for ligand-independent HH/GLI-activated tumors.

## Methods

### Reagents

PI3K inhibitor, LY294002, and KAAD-cyclopamine were purchased from Merck KGaA (Dharmstadt, Germany). Cell culture reagents and media were obtained from Invitrogen (Carlsbad, CA).

### Establishment of stable cell lines of A549 harboring GLI regulatory reporter gene

A549 cells were transfected with pLenti-bsd/GLI-bla vector (Invitrogen), which contains *β-lactamase *reporter gene under the transcriptional control of a (8×) Gli response element with tata-minimal promoter, by lipofection using Lipofectamine reagent (Invitrogen). The cells transfected with the vector were cultured with growth medium containing 3.8 μg/ml of blasticidin for 14 days, and stable cell lines were established. Several stable clones were transfected with *GLI1 *siRNA and a few clones were identified in which β-lactamase activity was reduced by more than 70% with *GLI1*-silencing.

### siRNA transfection and measurement of mRNA expression

The sequence of *p70S6K2 *siRNA used in the present experiments, other than kinome-wide siRNA screen, was GCCUAGAGCCUGUGGGACAtt (B-bridge, Mountain View, CA). The phenotypes observed in the p70S6K2 were confirmed by two additional sequences, GGUGUUCCAGGUGCGAAAGtt (Applied Biosystems/Ambion, Austin, TX) and GCAGAGAACCGGAAGAAAAtt (B-bridge). The following siRNAs were also used: *GLI1*-siRNA (M-003896-00-0020: Thermo Fisher Scientific Inc., Waltham, MA); *polo-like kinase 1 *(*PLK1*)-siRNA (M-003290-01-0010: Thermo Fisher Scientific Inc.); control-siRNA (D-001810-01-05: Thermo Fisher Scientific Inc.); and, human kinome siRNA set (AM80010V3, Applied Biosystems/Ambion). For siRNA transfection, 900 cells were seeded per well in 96-well plate and incubated for 24 hr. siRNA was mixed with a lipofection reagent, siLentFect (Bio-Rad, Hercules, CA) according to the manufacturer's instructions, and transfected into the A549 cells. mRNA was recovered and extracted 48 hr after transfection with RNAeasy (Qiagen, Hilden, Germany). Reverse transcription was performed for 500 ng of total RNA, and the cDNA obtained was applied to TaqMan PCR for quantification of mRNA expression. The primers and probe used for the quantitative polymerase chain reaction (qPCR) were: *p70S6K2 *(Hs00177689-M1, Applied Biosystems, Foster City, CA); *GLI1 *(Hs00171790-M1, Applied Biosystems), *Cyclin D1 *(Hokkaido system science, Sapporo, Japan); and, *γ-catenin *(Hs00158408-M1, Applied Biosystems). Data were collected and analyzed using an ABI 7900HT Fast Real-Time PCR System (Applied Biosystems). The relative mRNA expression data were normalized to *β-actin *expression, measured with pre-designed qPCR primers and probe (4310881E, Applied Biosystems).

### Cell viability assay and β-lactamase assay

Cell viability was measured by CellTiter-Glo Luminescent Cell Viability Assay (Promega, Madison, WI), 72 or 96 hr after siRNA transfection. An equal volume (100 μL) of CellTiter-Glo Reagent was added to medium, and mixed gently for 2 min on an orbital shaker. The solution was incubated at room temperature for 10 min to allow it to stabilize and luminescence to appear, after which the luminescence was measured. The activity of β-lactamase was quantified with GeneBLAzer™ Detection Kits (Invitrogen) according to the manufacturer's instructions. A 6 × substrate loading solution was added to the cells to 1 × final concentration and the cells in the buffer were incubated for 6 hr. β-lactamase activity was then measured using a fluorescent plate reader. The β-lactamase activity was normalized to cell number, measured by CellTiterGlo Luminescent Cell Viability Assay (Promega).

### Immunoblotting

For immunoblotting of total and phosphorylated GSK3β and GLI1, cell lysate was extracted from A549 or H1915 cells with a lysis buffer (50 mM HEPES, 250 mM NaCl, 0.1% NP-40, 0.1 mM DTT) comprising a 1:00 dilution of protease inhibitor cocktail (Thermo Fisher Scientific Inc. Rockford, IL) containing AEBSF, Aprotinin, Bestatin, E-64, Leupeptin, Pepstatin A), and a 1:00 dilution of phosphatase inhibitor cocktail (Thermo Fisher Scientific Inc.) containing sodium fluoride, sodium orthovanadate, sodium pyrophosphate and β-glycerophosphate. The extracted 20 μg of total protein was subjected to 10% SDS-PAGE analysis. Proteins were visualized by ECL chemiluminescence reagents (GE Healthcare UK Ltd., Buckinghamshire, UK) using primary antibodies specific to total GSK3β (#9315, Cell Signaling Technology, Danvers, MA), phosphorylated GSK3β at Ser9 residue (#9336, Cell Signaling Technology) and GLI1 (#2553, Cell Signaling Technology), p70S6K1 (#9202, Cell Signaling Technology) and p70S6K2 (sc-9379, Santa Cruz Biotechnology, Santa Cruz, CA).

## Competing interests

The authors declare that they have no competing interests.

## Authors' contributions

SM was involved in the design and execution of the experiments and drafted the manuscript. AK conducted most of the experiments and contributed to manuscript preparation. HH contributed to the overall experimental design. All authors read and approved the final manuscript.
